# Epigenetic Deregulation of Telomere-Related Genes in Newly Diagnosed Multiple Myeloma Patients

**DOI:** 10.3390/cancers13246348

**Published:** 2021-12-17

**Authors:** Samrat Roy Choudhury, Cody Ashby, Fenghuang Zhan, Frits van Rhee

**Affiliations:** 1 Pediatric Hematology-Oncology, Arkansas Children’s Research Institute, University of Arkansas for Medical Sciences, Little Rock, AR 72202, USA; 2 Department of Biomedical Informatics, University of Arkansas for Medical Sciences, Little Rock, AR 72205, USA; TCAshby@uams.edu; 3 Myeloma Center, Department of Internal Medicine, University of Arkansas for Medical Sciences, Little Rock, AR 72205, USA; FZhan@uams.edu (F.Z.); vanrheefrits@uams.edu (F.v.R.)

**Keywords:** Multiple Myeloma, DNA methylation, chromatin modifications, telomere-related genes, epigenetic biomarkers

## Abstract

**Simple Summary:**

Aberrant expression of telomere-related genes (TRGs) and telomere-shortening facilitates development of different types of cancer. Given the fact that high-risk multiple myeloma (MM) patients harbor critically shortened telomeres, we investigated the epigenetic basis of TRG-expression in newly diagnosed MM patients. We demonstrated that DNA methylation alone or in cooperation with overlapping chromatin marks and underlying genomic sequence build up an epigenetic network of aberrant gene expression in TRGs. Furthermore, we identified five TRGs, which are epigenetically controlled and consistently deregulated across the major molecular subgroups of MM, identifiable at the early stage of the disease.

**Abstract:**

High-risk Multiple Myeloma (MM) patients were found to maintain telomere length (TL), below the margin of short critical length, consistent with proactive overexpression of telomerase. Previously, DNA methylation has been shown as a determinant of telomere-related gene (TRG) expression and TL to assess risk in different types of cancer. We mapped genome-wide DNA methylation in a cohort of newly diagnosed MM (NDMM; *n* = 53) patients of major molecular subgroups, compared to age-matched healthy donors (*n* = 4). Differential methylation and expression at TRG-loci were analyzed in combination with overlapping chromatin marks and underlying DNA-sequences. We observed a strong correlation (R^2^ ≥ 0.5) between DNA methylation and expression amongst selective TRGs, such that demethylation at the promoters of *DDX1* and *TERF1* were associated to their oncogenic upregulation, while demethylation at the bodies of two key tumor suppressors *ZNF2*0*8* and *RAP1A* led to downregulation of the genes. We demonstrated that TRG expression may be controlled by DNA methylation alone or in cooperation with chromatin modifications or CCCTC-binding factor at the regulatory regions. Additionally, we showed that hypomethylated DMRs of TRGs in NDMM are stabilized with G-quadruplex forming sequences, suggesting a crucial role of these epigenetically vulnerable loci in MM pathogenesis. We have identified a panel of five TRGs, which are epigenetically deregulated in NDMM patients and may serve as early detection biomarkers or therapeutic targets in the disease.

## 1. Background

Multiple Myeloma (MM) is a B-cell neoplasm, manifested with abnormal infiltration of plasma cells (PCs) in the bone marrow matrix, which leads to the development of lytic bone lesions and myelosuppression [1]. The underlying polymorphic genetic and epigenetic variants in tumor cells strongly influence MM development and survival outcome [2,3,4,5]. In addition to etiological genetic and epigenetic features, telomere length (TL) has been identified as a crucial determinant of survival in patients with MM. 

Telomeres are TTAGGG-repeat enriched nucleoprotein complexes positioned at the termini of eukaryotic chromosomes and contribute to the maintenance of genomic stability and controlled cell-proliferation [6]. In contrast, cancer cells typically maintain shortened telomeres, favored by proactive catalytic activities of defective telomerase or through an alternative lengthening of telomere mechanism [7,8,9]. Previous studies have demonstrated a strong correlation between the shortening in leukocyte telomere length and the predisposition of developing different types of cancer, including hematological malignancies [10,11,12,13]. Particularly, patients with MM harboring high telomerase activity and short TL were marked as a ‘high-risk’ group, having significantly (*p* < 0.0001; hazard ratio = 3.4) shorter overall survival [14,15]. A progressive shortening in TL was also observed in patients with MM when compared to the patients at the stage defined as premalignant monoclonal gammopathy of undetermined significance (MGUS) [16]. Thus, an increased number of telomere aggregates were ascribed as a prognostic biomarker for high-risk in the smoldering multiple myeloma (SMM) patients, consistent with their poor drug susceptibility and short-term survival [17]. While TL maintenance is found to be crucially linked to the prognosis and survival of the disease, both long- and short-term inhibition of telomerase resulted in reduced growth of MM cells [18,19].

The regulation of telomerase activity and the maintenance of TL in MM could be influenced by a set of extrinsic and intrinsic factors. For instance, cytokines such as interleukin 6 and insulin-like growth factor 1 were reported to increase telomerase activity in MM cells without alterations to the human telomerase encoding gene, telomerase reverse transcriptase (*hTERT*) [20]. In contrast, bortezomib, the anticancer medication used to treat MM, consistently reduced both *hTERT* expression and telomerase activity in MM cell lines [21]. Intrinsic factors such as genetic mutations or DNA methylation at subtelomeres and telomere-related genes (TRGs) were also reported to have a strong association with TL maintenance in different types of cancer [22,23,24,25,26]. A few previous studies have attempted to establish the relationship between differential expression of TRGs and TL maintenance in MM [27,28]. However, to our knowledge, no previous study had been reported to investigate the epigenetic basis of differential TRG expression in the disease.

To address this deficiency, we have combined epigenetic modifications, such as DNA methylation and chromatin marks, to determine their impact on the expression of a complete catalog of TRGs [29]. For the present study, we selected a cohort of patients with NDMM belonging to six major molecular subgroups, categorized based on immunoglobulin heavy chain locus (*IgH*) translocation (TC) with a number of oncogenes such as *FGFR3/MMSET*, t(4;14); *CCND1*, t(11;14); *MAF*, t(14;16); or *MAFB*, t(14;20); and non-TC hyperdiploid (HY) subgroups with oncogenic overexpression of multiple copies of *CCND1* (D1) or *CCND2* (D2). We also investigated overlaps of G-quadruplex forming sequences (G4FS) to the epigenetic risk-loci spanning the TRGs in patients with NDMM and in representative MM cell lines.

## 2. Methods

### 2.1. Sample Information and Genomic DNA/RNA Preparation

After approval by the institutional review board, CD138-positive bone marrow PCs were collected from a cohort of consented patients with NDMM (*n* = 53) and compared to pools of age-matched healthy donors (controls; *n* = 4). Bone marrow aspirates were enriched for >90% tumor cells using RoboSep automated cell separation techniques (Stemcell Technologies, Vancouver, British Columbia, Canada). We included patients with NDMM from the t(4;14), *n* = 8; t(11;14), *n* = 10; t(14;16), *n* = 9; and t(14;20), *n* = 7, TC subgroups and the D1, *n* = 12, and D2, *n* = 7, HY subgroups. Demographic information of the enrolled subjects is summarized in Table S1. We have also included MM cell lines, representative of the major *IgH* translocations found in MM patients, such as H929 for t(4;14), U266 for t(11;14), MM.1S for t(14;16), and SACHI for t(14;20) subgroups. MM cell lines were maintained in RPMI 1640 with 20% fetal bovine serum, 1% L-glutamine, and 1% penicillin-streptomycin. Authenticity of the cell lines were checked with short tandem repeat analysis of non-coding DNA, while possibilities of microbial contaminations were nullified with mycoplasma check. Genomic DNA and RNA samples were extracted using AllPrep DNA/RNA/Protein Mini Kit (Qiagen, Hilden, Germany) or Puregene DNA Extraction Kit (Qiagen, Hilden, Germany).

### 2.2. Library Preparation and Sequence Annotations for Reduced Representation Bisulfite Sequencing

We prepared libraries for reduced representation bisulfite sequencing (RRBS), following our previously published protocol [30]. Briefly, 100 ng of genomic DNA from MM cell lines or patient-derived samples were digested with MspI overnight to generate ~5-kb fragments along the enzyme cut sites across genome. Next, we performed the end-repair and A-tailing of DNA fragments using the KAPA HyperPlus Kit (Roche, Basel, Switzerland) followed by ligation of methylated adaptors (NEBNext Multiplex Oligos for Illumina; Methylated Adaptor, Index Primers Set; New England BioLabs Inc., Ipswich, MA, USA), bisulfite-conversion (EZ DNA Methylation Kit; Zymo Research, Irvine, CA, USA), and amplification of the barcoded libraries (EpiMark Hot Start *Taq* DNA Polymerase, New England BioLabs Inc., Ipswich, MA, USA). RRBS libraries were then selected based on fragment sizes of 150–250 bp and 250–450 bp. Quality control of the libraries was performed by determining the concentration, using a high-sensitivity DNA assay kit on the Qubit spectrometer (Thermo-Fisher, Waltham, MA, USA), and for size, using the 2200 TapeStation System (Agilent Technologies, Santa Clara, CA, USA), before sequencing with 75 bp single-end reads on the NextSeq 500 System (Illumina, San Diego, CA, USA). Genome sequencing of the raw data (binary alignment/map files) was processed, filtered, and the genome (hg38/GRCh38) annotated using our previously published guidelines [3]. Briefly, differentially methylated regions (DMRs) were annotated by identifying the closest transcription start site (TSS) of a gene based on the National Center for Biotechnology Information Reference Sequence Database. DMRs located from 5 kb upstream to 200 bp downstream of TSS were marked as the promoters, while DMRs spanning up to the 3′-end of the corresponding genes were marked as the body. DMRs having a methylation difference (at a false discovery rate [FDR]-adjusted *p* value < 0.05) of >20% increase (hypermethylation) or decrease (hypomethylation) between NDMM subgroups and controls) were considered significant for all the downstream analyses. The difference of mean DMR-methylation (MDM) at the promoters and the bodies were determined in NDMM subgroups and MM cell lines in comparison to control.

### 2.3. Gene Expression Analysis

Gene expression profiling was performed using Affymetrix GeneChip Human Genome U133 Plus 2.0 Array (Thermo-Fisher, Waltham, MA, USA). The raw files were processed with Transcriptome Analysis Console v4.0 Software (Thermo-Fisher, Waltham, MA, USA). The signal intensity values of multiple transcripts (whenever applicable) were averaged, while the difference in the median expression of samples between NDMM subgroups and control less than 1-fold with adjusted *p* < 0.05 (using log2 scale) were filtered out. Venn intersection analyses were performed using Venny v2.1 (BioinfoGP, Madrid, Spain) to determine the genes that have an inverse methylation-expression relationship at the promoters or a direct methylation-expression relationship at the bodies. A regression analysis was performed to determine the coefficient of correlation (R^2^) between DNA methylation and expression.

### 2.4. Analysis of Chromatin Modifications

We have analyzed the enrichment of four activating histones (H3K27ac, H3K4me1, H3K4me3, and H3K36me3) and one repressive histone (H3K27me3), overlapped to DMRs of the differentially expressed TRGs using chromatin immunoprecipitation sequencing (ChIP-seq) in reference to H929 t(4;14) MM cells. The enrichment of CCCTC binding factor (CTCF)-transcription factor (TF) to the overlapping DMRs was also determined in reference to the ChIP-seq data in H929 cell line. Additionally, open chromatin states on or adjacent to the DMRs were determined with DNAse sequencing (DNAse-seq). Both the ChIP-seq and DNAse-seq data were obtained from the Encyclopedia of DNA Elements (ENCODE) epigenomics data (as uploaded by the Noble Laboratory at University of Washington using avocado:encode2018core) for H929 cells [31]. The chromatin enrichment and position of DMRs were visualized with the Integrated Genomics Viewer (IGV), followed by loading bigwig and customized bed files onto the IGV respectively [32].

### 2.5. Determine G-Quadruplex–DMR Overlaps

The G4FS, overlapped with the DMRs of TRGs, were analyzed using a web-based server that predicts quadruplex forming G-rich sequences (QGRS), the QGRS Mapper [33].

### 2.6. Statistical Analysis

Methylation and expression data were prepared and presented with Prism v8.0 software (GraphPad, San Diego, CA, USA). A two-way ANOVA test was used to compare pair-wise differential methylation at DMRs between NDMM subgroups and controls. A one-way ANOVA with multiple comparison was performed for expression analysis, and an adjusted *p* value < 0.05 was considered significant.

## 3. Results

### 3.1. Selective TRGs Are Differentially Expressed in NDMM

**Figure 1:**
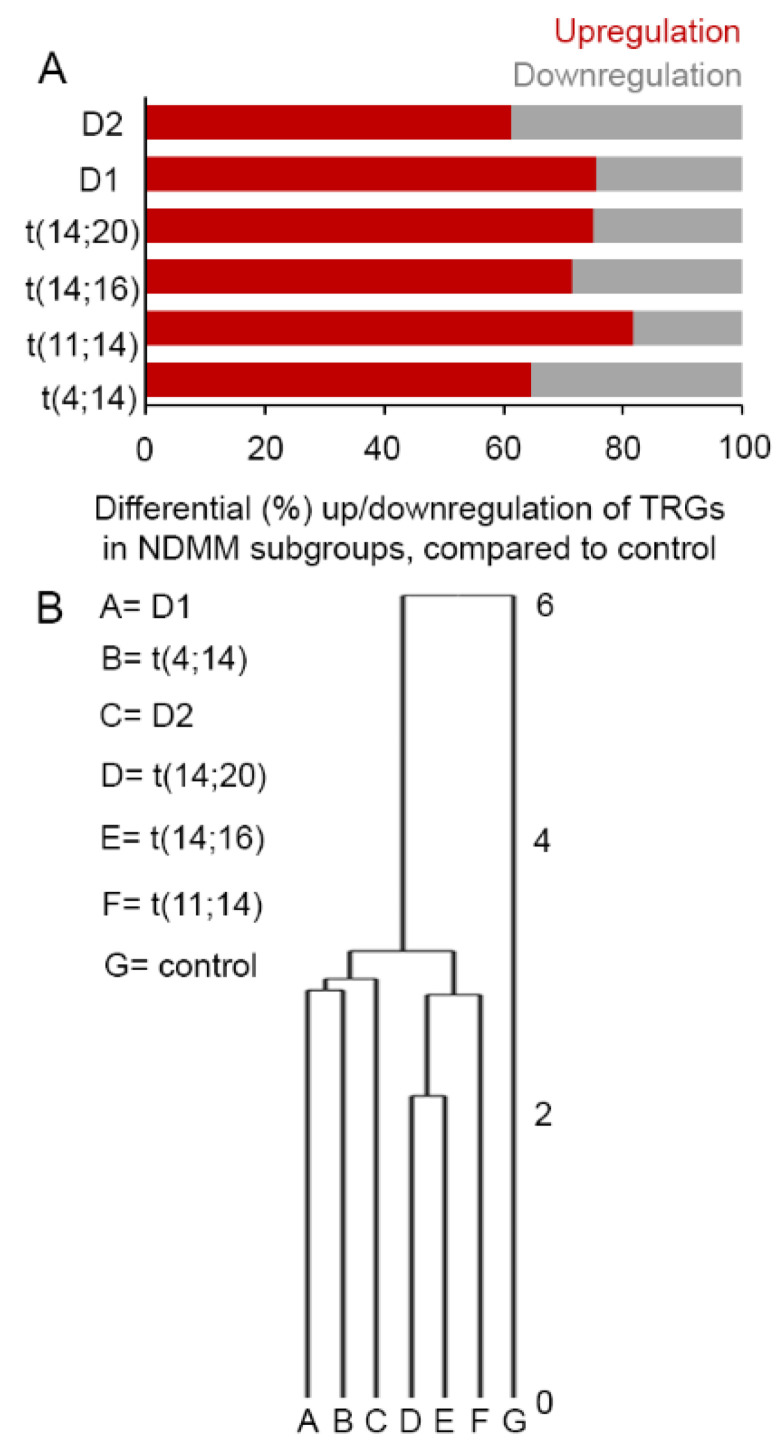
(**A**) A subgroup-specific differential telomere-related genes (TRG)s expression pattern was seen in major molecular subgroups of newly diagnosed multiple myeloma (NDMM) patients compared to controls. (**B**) Unsupervised hierarchical clustering with differentially expressed TRGs showed distinct clustering between D1 and t(4;14) subgroups or MAF subgroups containing t(14;16) or t(14;20) translocations. Abbreviation: NDMM; newly diagnosed multiple myeloma.

First, we assessed differentially expressed TRGs (NDMM subgroups vs. control), which are involved in a multitude of intrinsic biological pathways including helicase, telomerase, shelterin-complex, DNA-repair, or related biosynthetic activities, as summarized elsewhere [29]. Differential expression of epigenetically regulated TRGs have also been reported in relation to their importance to the development of different types of cancer [34,35]. We aimed to evaluate the impact of 70 known TRGs in MM and observed a subgroup-specific differential expression pattern among the NDMM subgroups. [29]. For instance, we observed 65% (40/62) upregulated and 35% (22/62) downregulated TRGs in t(4;14), 82% (49/60) upregulated and 18% (11/60) downregulated TRGs in t(11;14), 71% (45/63) upregulated and 29% (19/63) downregulated TRGs in t(14;16), 75% (45/60) upregulated and 25% (15/60) downregulated TRGs in t(14;20), 75% (43/57) upregulated and 25% (14/57) downregulated TRGs in D1, and 61% (38/62) upregulated and 39% (24/62) downregulated TRGs in D2 subgroup (Figure 1A, Table S2). An unsupervised hierarchical clustering based on TRG-expression in NDMM subgroups revealed closest neighboring clusters between D1 and t(4;14) and between t(14;16) and t(14;20). The D2 remains equidistant to D1 and t(4;14), while t(11;14) remains equidistant to the t(14;16) and t(14;20) subgroups. In contrast, the control group formed a significantly distant cluster to all the NDMM subgroups (Figure 1B).

**Figure 2:**
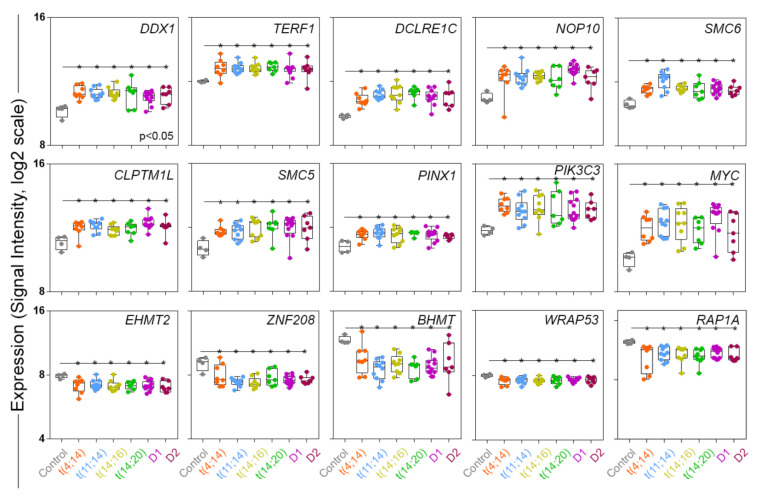
Selective TRGs are significantly (*p* < 0.05) differentially upregulated (*n* = 10) or downregulated (*n* = 5) across major *IgH* translocation (*n* = 4) and hyperdiploidy (*n* = 2) subgroups in a cohort of patients with NDMM, compared to control. Abbreviations: *IgH*, immunoglobulin heavy chain locus; NDMM, newly diagnosed multiple myeloma; TRG, telomere-related gene.

Next, we narrowed down a panel of TRGs, based on their significant (*p* < 0.05) and consistent differential expression across the NDMM subgroups compared to control. For instance, we observed an upregulation of helicase associated DEAD-box helicase 1, *DDX1* (mean differential expression [DE] between NDMM subgroups and control, DE: 0.75, log2); shelterin-bound telomerase repeat binding factor 1, *TERF1* (DE: 1.2, log2); NOP10 ribonucleoprotein, *NOP1*0 (DE: 1.42, log2); DNA-repair related DNA cross-link repair 1C, *DCLRE1C* (DE: 1.32, log2); structural maintenance of chromosomes 5 and 6, *SMC5* (DE: 1.73, log2) and *SMC6* (DE: 1.34, log2); and other biological process related TRGs such as CLPTM1-like, *CLPTM1L* (DE: 0.87, log2); *PIN2* (TERF1) interacting telomerase inhibitor 1, *PINX1* (DE: 0.96, log2); phosphatidylinositol 3-kinase catalytic subunit type 3, *PIK3C3* (DE: 0.61, log2); and MYC Proto-Oncogene, *MYC* (DE: 1.89, log2). In contrast, we observed downregulation of five TRGs across the NDMM subgroups, compared to control. For example, shelterin bound Ras-related protein 1A, *RAP1A* (DE: −0.7, log2); telomerase-associated WD repeat containing antisense to TP53, *WRAP53* (DE: −0.37, log2); other TRGs such as euchromatic histone lysine methyltransferase 2, *EHMT2* (DE: −0.9, log2); betaine-homocysteine S-methyltransferase, *BHMT1* (DE: 0.79, log2); and zinc finger protein 208, *ZNF2*0*8* (DE: −0.6, log2) were downregulated (Figure 2).

In summary, while TRG expression was typically variable across molecular NDMM subgroups, a cluster of TRGs was consistently differentially expressed in the major molecular subgroups of NDMM discussed here.

### 3.2. Selective TRGs Are Differentially Methylated and Tightly Correlated to Expression in NDMM

**Figure 3:**
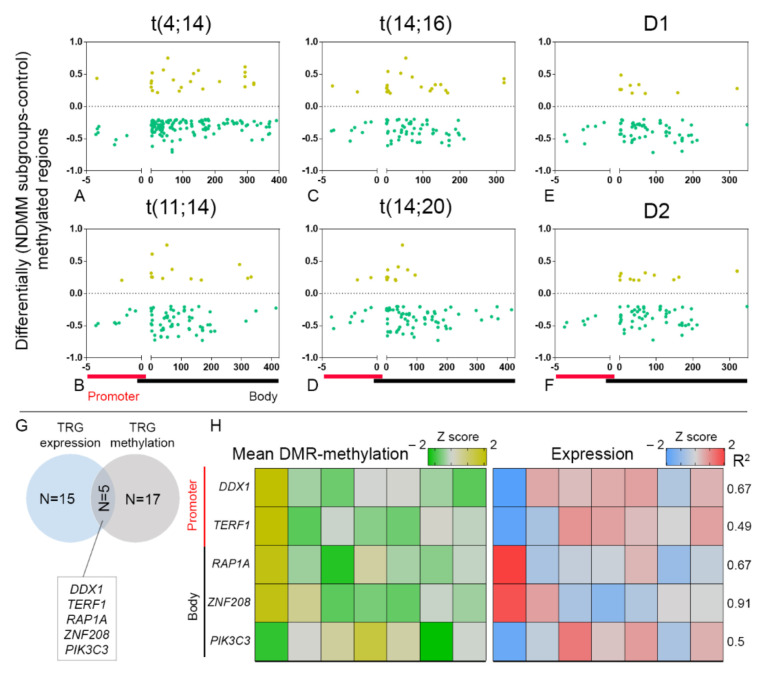
(**A**–**F**) Distribution of hypermethylated and hypomethylated DMRs across the promoters (−5 kb of TSS) and bodies (up to 3′ UTR) of TRGs among NDMM subgroups. (**G**) Out of 15 significantly (adj *p* < 0.05) differentially expressed and 17 differentially (*p* < 0.05) methylated TRGs among NDMM subgroups, we observed 16% (*n* = 5) of TRGs that were mutually inclusive to both the categories. (**H**) We observed strong correlation (R^2^ ≥ 0.5) between MDM and expression in 5 TRGs, where an inverse methylation-expression at the promoter, or direct methylation-expression relationship at the body were considered. Abbreviations: DMR; differentially methylated regions; MDM, mean DMR-methylation; NDMM, newly diagnosed multiple myeloma; TRG, telomere-related gene; TSS, Transcription Start Site.

We analyzed significant DMRs (*p* < 0.05 differential methylation greater or less than 20% between NDMM and controls) of 70 known TRGs across their promoters and bodies as detected per NDMM subgroups. The gene bodies were found to have multiple DMRs of a representative gene, while TRGs at the promoters were typically represented with single DMRs. In general, the majority of DMRs in both the genomic regions were found hypomethylated across the subgroups. For instance, we observed 12% hypermethylated (1/8) and 88% hypomethylated (7/8) DMRs representing six unique TRGs at the promoters and 14% hypermethylated (21/146) and 86% hypomethylated (125/146) DMRs at the bodies, representing 30 unique TRGs in NDMM subgroup t(4;14) (Figure 3A, Table S3).

In t(11;14) subgroup, we observed 11% hypermethylated (1/9) and 89% hypomethylated (8/9) DMRs at the promoters, representing 9 unique TRGs and 16% hypermethylated (11/69) and 84% hypomethylated (58/69) DMRs at the bodies representing 18 unique TRGs (Figure 3B and Table S4). The t(14;16) and t(14;20) subgroups showed high similarities in number and distribution of DMRs across the promoters, having 20% hypermethylated (2/10) and 80% hypomethylated (8/10) DMRs, representing 10 unique TRGs. In contrast, subtle disparities were observed in DMR distribution at the bodies among the subgroups with 26% hypermethylated (18/70) and 74% hypomethylated (52/70) DMRs of the t(14;16) subgroup, representing 20 unique TRGs, and 13% hypermethylated (10/78) and 87% hypomethylated (68/78) DMRs of the t(14;20) subgroup, representing 23 unique TRGs (Figure 3C,D, Tables S5 and S6). Among HY, we observed 100% hypomethylated (7 in D1; 6 in D2) DMRs (also unique TRGs) in the promoters. In D1 subgroup, 13% hypermethylated (8/61) and 87% hypomethylated (53/61) DMRs were observed in the bodies representing 18 unique TRGs, while in D2, 18% hypermethylated (11/64) and 83% hypomethylated (53/64) DMRs were observed in the bodies representing 19 unique TRGs (Figure 3E,F, Tables S7 and S8).

Next, we focused on the TRGs that were differentially methylated in all the NDMM subgroups compared to control, and documented five TRGs at the promoters (such as *DDX1, RAD54L, TP53BP1, MTR, and TERF1*) and 12 TRGs at the bodies (*ACYP2, DDB1, MAD1L1, MEN1, PIK3C3, PRMT8, RAP1A, RECQL4, RECQL5, RTEL1, TERT,* and *ZNF2*0*8*). A subsequent intersection analysis containing differentially methylated and expressed TRGs among the subgroups revealed five TRGs (*DDX1, PIK3C3, RAP1A, TERF1,* and *ZNF2*0*8*) (Figure 3G), where expression is dependent on DNA-methylation. Next, we evaluated the correlation between methylation and expression of these genes and observed a strong negative correlation (R^2^) between methylation and expression for the promoter DMRs, as observed for TRGs such as *DDX1* (R^2^ = 0.67) and *TERF1* (R^2^ = 0.49). In contrast, strong positive correlations were observed between methylation and expression for the body DMRs representing TRGs such that *RAP1A* (R^2^ = 0.67) and *ZNF2*0*8* (R^2^ = 0.91) were downregulated in accordance with hypomethylation, while *PIK3C3* (R^2^ = 0.5) was upregulated in accordance with increase in methylation (Figure 3H). In summary, we demonstrated a robust correlation between DNA methylation and expression of selective TRGs, evident in the early stage of the disease.

### 3.3. TRG-Methylation Is Fairly Conserved between Patients with NDMM and MM Cell Lines

**Figure 4:**
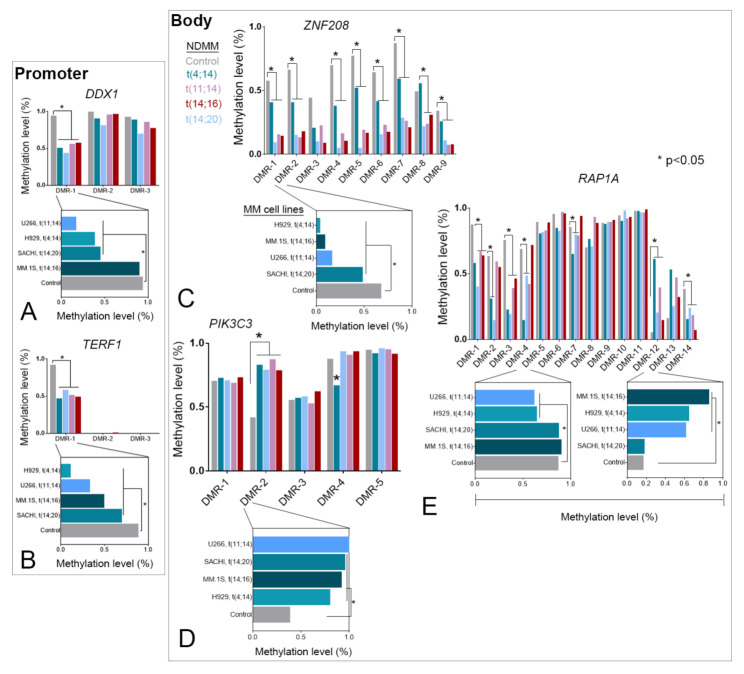
We observed similarities in DNA methylation pattern across DMRs of representative TRGs between NDMM subgroups and MM cell lines. For instance, DMR-1 at the promoters of (**A**) *DDX1* and (**B**) *TERF1*, and DMRs at the bodies of (**C**) *ZNF208*, and (**E**) *RAP1A* were hypomethylated. We observed a single (DMR-2; *p* < 0.05) hypermethylated DMR at the body of *PIK3C3* (**D**). Abbreviations: DMR, differentially methylated regions; NDMM, newly diagnosed multiple myeloma; TRG, telomere-related gene.

Epigenetic polymorphisms (including DNA methylation) among patients may significantly influence variations in therapeutic response and survival outcome [36,37]. CpG sites (cytosine and guanine occurring consecutively) that are crucial to the development or maintenance of a disease are typically well-conserved between patients and cell lines [38]. To investigate the importance of the TRG-DMRs, we further compared the DNA methylation level and distribution along DMRs between TC NDMM subgroups and representative MM cell lines. We compared the MDM across NDMM subgroups and MM cell lines compared to control. For example, DMR-1 (MDM = −0.42, *p* = 0.001 in NDMM; MDM = −0.46, *p* = 0.038 in MM lines) of *DDX1* and DMR-1 (MDM = −0.4, *p* = 0.026 in NDMM; MDM = −0.47, *p* = 0.019 in MM lines) of *TERF1* promoters were hypomethylated in both patients and cell lines (Figure 4A,B). Of nine DMRs at the body of *ZNF2*0*8* in the NDMM subgroups, only DMR-2 was found consistently hypomethylated between patients (MDM = −0.44, *p* = 2.09 × 10^−8^) and cell lines (MDM = −0.48, *p* = 0.0411) (Figure 4C).

In contrast, DMR-2 of *PIK3C3* was consistently hypermethylated in patients (MDM = 0.4, *p* = 0.0016) and cell lines (MDM = 0.53, *p* = 0.0022) (Figure 4D). For *RAP1A,* we observed 7 out of 14 significantly (*p* < 0.05) methylated DMRs in NDMM subgroups, of which DMR-1 through DMR-4 and DMR-14 were hypomethylated, and DMR-12 was hypermethylated (Figure 4E). However, we noticed that in DMR-2, only t(4;14) and t(14;20) subgroups were significantly (*p* < 0.05) hypomethylated compared to control. Another interesting observation from *RAP1A* was the disparity in methylation between DMRs in NDMM subgroups and MM cell lines, such that DMR-5 was not differentially methylated in NDMM subgroups (MDM = −0.05, *p* = 0.593), but was hypomethylated in MM cell lines (MDM = −0.21, *p* = 0.005). Additionally, compared to hypermethylation at DMR-12 in patients, DMR-13 was particularly hypermethylated in cell lines (MDM = 0.4, *p* = 0.0007). Noteworthy, TRGs were also consistently upregulated or downregulated between patients and cell lines. Cumulatively, we observed considerable similarities in the DNA methylation profile at selective DMRs that were consistent between NDMM subgroups and MM cell lines with few above-mentioned exceptions.

### 3.4. DNA Methylation Alone or Cooperatively with Chromatin Marks Regulates TRG Expression

**Figure 5:**
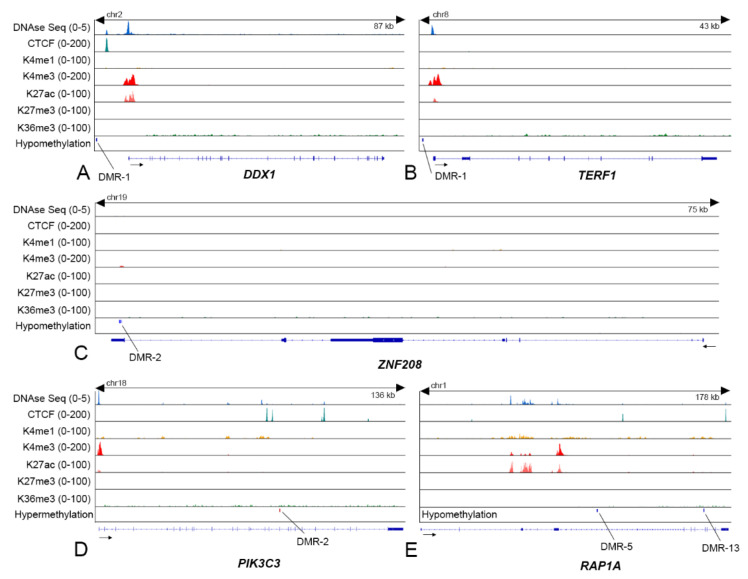
Proximity and possible overlaps between DMRs and chromatin marks were mapped on selective epigenetically regulated TRGs (*n* = 5). ChIP-sequencing data from H929, t(4;14) cell line containing four activating histones (H3K4me1, H3K4me3, H3k27ac, and H3K36me3), and one repressive histone (H3K27me3) were mapped to the proximal DMRs. Additionally, DNAse-seq peaks were observed to determine open chromatin, while CTCF binding was also observed. The overlaps between DNA and chromatin level epigenetic marks were visualized at 5 kb upstream TSS of (**A**) *DDX1* and (**B**) *TERF1*, or across the body region of (**C**) *ZNF208*, (**D**) *PIK3C3*, and (**E**) *RAP1A*. Abbreviations: ChIP, chromatin immunoprecipitation; CTCF, CCCTC binding factor; DMR, differentially methylated regions; DNAse-seq, DNAse sequencing; TRG, telomere-related gene; TSS, transcription start site.

We wanted to determine if the expression of TRGs was controlled by DNA methylation alone or in cooperation with other epigenetic factors, such as histone marks, TF binding, or chromatin state. Given the consistencies in DNA methylation across DMRs between NDMM subgroups and MM cell lines, we investigated the chromatin marks in the MM cell line H929, t(4;14) in reference to the DMRs of the NDMM patient-subgroups. We mapped proximities or overlaps between histone marks, CTCF binding sites, and open chromatin state to selective TRG-DMRs. For instance, a DMR (DMR-1 of *DDX1*) spanning base pairs 15,586,861 to 15,587,037 on chromosome 2 (chr2:15586861 to chr2:15587037) at 4.4 kb upstream of the TSS of *DDX1*, and a DMR (DMR-1 of *TERF1*) spanning chr8:73007342 to chr8:73007540 at 1.3 kb upstream of the TSS of *TERF1* contained an abundance of activating histone H3K4me3 and relatively lesser enrichment for H3K27ac (Figure 5A,B). The chromatin adjacent to DMRs of both the genes was found in an open state, as evidenced from DNAse peaks. Additionally, a sharp CTCF peak was observed at the upstream TSS of *DDX1*. Hypomethylation at the promoters, coincident with activating histone marks and open chromatin state, explained upregulation of *DDX1* (DE: 1.2, log2) and *TERF1* (DE: 1.2, log2).

In contrast, hypomethylation observed at DMR-2 spanning chr19:21940986 to chr19:21941044, DMR-5 along the body of *ZNF2*0*8* spanning chr1:111608116 to chr1:111608192, and DMR-13 spanning chr1:111689837 to chr1:111689970 across *RAP1A* gene-body were consistent with downregulation of *ZNF2*0*8* (DE: −0.6, log2); and *RAP1A* (DE: −0.7, log2) (Figure 5C,E). While no prominent chromatin marks were found across the promoter or the body of *ZNF2*0*8*, subtle histone enrichments and open chromatin loci were observed in the body of *RAP1A*; however, were considerably distant (>5 kb) to the DMRs. In these two examples, DNA methylation is likely to control TRG expression, independently or with chromatin modifications. The DMR-2 spanning chr18:42024964 to chr18:42025106) of *PIK3C3* was hypermethylated and proximal (<5 kb) to CTCF binding sites, which is consistent with upregulation (DE: 0.61, log2) of the gene (Figure 5D). Cumulative data demonstrated variable epigenetic regulations, linked to the TRG expression in the disease.

### 3.5. TRG Hypomethylated DMRs Are Stabilized with G-Quadruplexes

**Figure 6:**
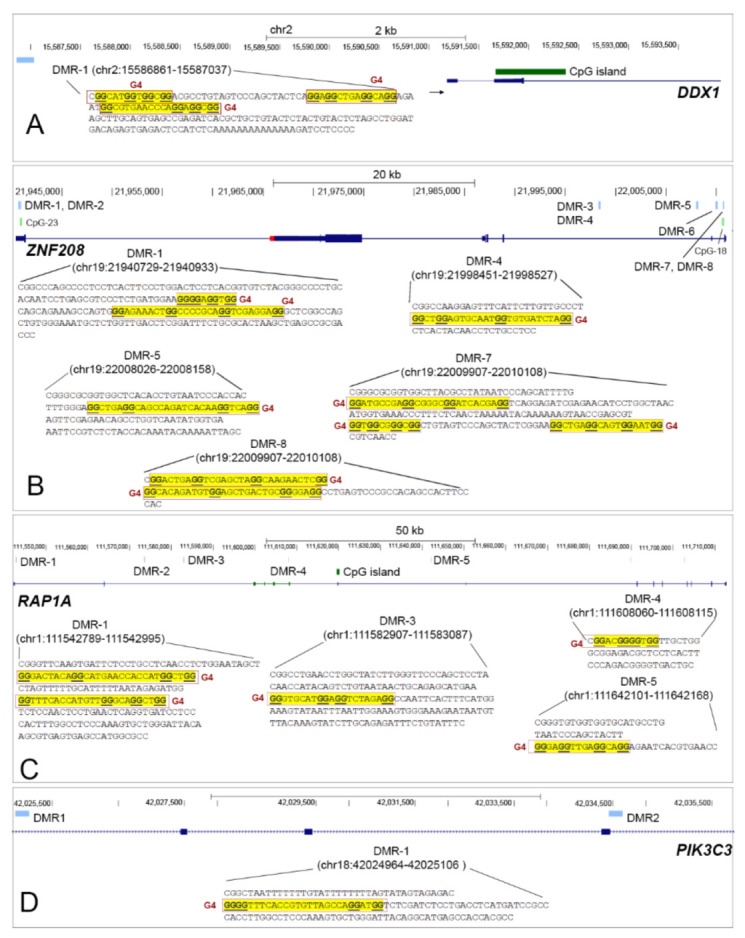
We observed frequent overlaps between hypomethylated DMRs and G4FS at (**A**) the promoter of *DDX1*, and the body of (**B**) *ZNF208* and (**C**) *RAP1A*. (**D**) A single G4FS was observed, overlapped to the hypermethylated DMR of *PIK3C3*, while no overlap was noticed at the DMR-1 of the *TERF1* promoter. Abbreviations: DMR, differentially methylated regions; G4FS, G quadruplex forming sequences.

Given the fact that aberrantly hypomethylated genomic regions, enriched for G4FS have significant contributions to the genomic instabilities in cancer [39], we investigated overlaps of G4FS with the DMRs of TRGs to understand the possible impact of these genetic–epigenetic variants on sustaining stability of these TRG-related epigenetic risk-loci in the disease. We observed three G4FS overlapped with the hypomethylated DMR-1 (177 bp) at the promoter of *DDX1* (Figure 6A), which also coincides to the open chromatin region.

However, no G4FS was found in the DMR-1 of *TERF1*. Nonetheless, similar to *DDX1*, frequent overlaps between G4FS and hypomethylated DMRs were found at the bodies of *ZNF2*0*8* and *RAP1A*. For instance, 56% (5/9) of DMRs at the *ZNF2*0*8* body were overlapped with G4FS, of which DMR-1 (205 bp) has two G4FS, DMR-4 (77 bp) has one G4FS, DMR-5 (133 bp) has one G4FS, DMR-7 (202 bp) has three G4FS, and DMR-8 has two G4FS (Figure 6B). For *RAP1A,* we observed 29% (4/14) overlap between G4FS and hypomethylated DMRs having two G4FS in DMR-1 (207 bp), one G4FS in DMR-3 (181 bp), one G4FS in DMR-4 (56 bp), and one G4FS in DMR-1 (143 bp) (Figure 6C). For *PIK3C3*, we observed a single G4FS, overlapped with the DMR-1 (143 bp), which was not significantly differently methylated across NDMM subgroups compared to control (Figure 6D). In summary, we have demonstrated that hypomethylated DMRs of selective TRGs are frequently stabilized by G4FS.

## 4. Discussion

To the best of our knowledge, this is the first study to report that DNA methylation alone, or in cooperation with chromatin state and histone marks, deregulate aberrant selective TRG expression in early-stage MM.

In general, the majority of TRGs in our NDMM cohort were differentially upregulated (81%) rather than downregulated (19%) across the subgroups. For instance, upregulation of the TRGs, such as *DKC1*, *GAR1*, *NHP2*, and *NOP1*0, were found in several NDMM subgroups (Table S2), which is consistent to the previous finding where expression of these genes was progressively increased along with the development from MGUS to the MM stage [27]. In contrast, *WRAP53*, which is known to bind the Cajal body box of the telomerase RNA template component, was downregulated (DE: −0.37, log2). Reduced expression of *WRAP53* has been previously correlated to attenuated DNA damage response and poor survival in ovarian and lung cancers [40,41], and suggests similar line of dysregulation in MM. Of the shelterin proteins, *TERF1* was upregulated, while *RAP1A* was downregulated, which suggest defunct telomere maintenance, favorable of progression of the disease. In particular, *TERF1* overexpression was found strongly correlated with TL and disease progression in gastric and renal carcinomas [42,43]. Another interesting finding from the TRG expression profile was the upregulation of 3 (*DCLRE1C, SMC5, and SMC6)* of 15 detected repair related TRGs in NDMM subgroups compared to control. DNA-repair genes have previously been found to be upregulated in response to chemotherapy and radiotherapy in a number of cancers including melanoma manifested with metastasis [44,45]. In summary, the TRG expression profile suggests an involvement of a compromised DNA-repair mechanism machinery, coincident with a dysfunctional telomere maintenance in the disease.

Next, we found a strong correlation (R^2^ ≥ 0.5) between DNA methylation and expression of five TRGs. In comparison to the previous studies, where DNA methylation changes at individual CpGs were correlated to changes in TRGs expression, we considered all significant DMRs along regulatory regions of the TRGs to determine the methylation–expression relationship in MM [29]. Moreover, we have investigated the role of differential methylation at both the promoters and the bodies of TRGs, considering a unique crucial role of the regulatory regions on gene expression [46,47]. We observed the promoter of *DDX1* and *TERF1* was hypomethylated, where DMRs of both genes were proximal to H3K4me3 and H3K27ac enriched TSS. This corroborates the fact that hypomethylation at the promoter functions cooperatively with activating chromatin marks to facilitate binding of TFs to upregulate these oncogenes. This seems more reliable given the appearance of DNAse peaks suggesting open chromatin state on the genes. Additionally, we observed CTCF binding to the *DDX1* promoter. This could be explained with colocalization of CTCF with the promoter bound H3K4me3 or H3K27ac regarding upregulation of the gene [48]. We also observed a strong positive correlation between DNA methylation and expression at the bodies, such that *RAP1A* and *ZNF2*0*8* were downregulated, consistent with hypomethylation across the body DMRs, while *PIK3C3* was upregulated, consistent with hypermethylation across the body DMRs. This finding is highly fascinating because aberrant upregulation of several oncogenic TRGs is complemented with epigenetic silencing of two key tumor suppressors, *RAP1A* and *ZNF2*0*8* [49,50,51]. Additionally, gene-body DNA methylation was found as a principal determinant of expression because DMRs were not overlapped or proximal (>5 kb) to any regulatory histone marks or CTCF binding sites at open chromatin regions. 

Our study provides a potential mechanistic insight of robust epigenetic control of TRG expression in patients with NDMM, which could be considered as potential early-detection biomarkers and therapeutic targets for the disease. The DMRs, as observed in the NDMM subgroups might also have a profound role in the progression of the disease, as we found several of these DMRs to be conserved between NDMM subgroups and MM cell lines, which represent a terminal differentiation stage of the disease. However, we found a relatively higher degree of hypermethylation and hypomethylation in the cell lines compared to the patient-derived samples. We also found a general trend of hypomethylation (except for *PIK3C3*) in TRG-DMRs, which were frequently overlapped with G4FS. Our findings are in alignment with previous findings that G4FS may stabilize and conserve CpG sites at hypomethylated states by inhibiting DNA methyltransferase 1 enzymatic activity, which in turn facilitates enhanced DNA breakpoints or chromatin accessibility [39,52,53].

In summary, we have identified a number of crucial epigenetic risk-loci that might play crucial roles in TRG dysfunctions and overall development of the disease. These epigenetic risk-loci are consistently deregulated in NDMM subgroups and detectable at the early stage of the disease. However, future study needs further expansion with inclusion of subjects from different disease stages (such as premalignant MGUS, transient SMM, and MM with patients from relapsed and refractory cohorts) to compare dynamics of epigenetic alterations at TRG loci. Finally, given the advancement in sequencing techniques to accurately match the DNA methylation profile of cell-free DNA to the tissues and cell types, it could serve as a noninvasive screening platform for epigenetic biomarkers in patients with MM [54].

## 5. Conclusions

We have identified a 5 TRG panels, tightly regulated with epigenetic modifications such as DNA methylation, chromatin modifications, or G4FS in patients with NDMM. Using clustered regularly interspaced short palindromic repeats (CRISPR) tools, it would be highly interesting to investigate whether conditional alterations in DNA methylation or chromatin marks on these TRGs could alter gene expression, TL and inhibit overall progression of the disease. Investigations of epigenetically deregulated TRG pathways are also expected to provide valuable information for developing novel chemotherapies and immunotherapies in MM.

## Data Availability

The datasets used and/or analyzed during the current study are available from the corresponding author and with permission of University of Arkansas for Medical Sciences on reasonable request.

## Supplementary Materials

The following are available online at https://www.mdpi.com/article/10.3390/cancers13246348/s1, Table S1: Demographic features of patients with NDMM, Table S2: Median expression of TRG transcripts in major molecular subgroups of patients with NDMM, Table S3: Differential methylation across the promoter and the body of TRGs in t(4;14) subgroup, Table S4: Differential methylation across promoter and body of TRGs in t(11;14) subgroup, Table S5: Differential methylation across promoter and body of TRGs in t(14;16) subgroup, Table S6: Differential methylation across promoter and body of TRGs in t(14;20) subgroup, Table S7: Differential methylation across promoter and body of TRGs in D1 subgroup, Table S8: Differential methylation across promoter and body of TRGs in D2 subgroup.

## Abbreviations

ChIP-seq	Chromatin immunoprecipitation sequencing
CTCF	CCCTC binding factor
DMR	Differentially methylated region
FC	Fold change
FDR	False discovery rate
G4FS	G-quadruplex forming sequences
*hTERT*	Telomerase reverse transcriptase encoding gene
HY	Hyperdiploidy
IgH	Immunoglobulin heavy chain locus
MDM	mean DMR methylation
MGUS	Monoclonal gammopathy of undetermined significance
MM	Multiple Myeloma
NDMM	Newly diagnosed multiple myeloma
QGRS	Quadraples forming G-rich sequences
RRBS	Reduced representation bisulfite sequencing
SMM	Smoldering multiple myeloma
TC	Translocation
TF	Transcription factor
TL	Telomere length
TRG	Telomere-related gene
TSS	Transcription start site
