# Supplementary material for: Epigenetic Deregulation of Telomere-Related Genes in Newly Diagnosed Multiple Myeloma Patients
Source: Cancers (Basel). 2021 Dec 17;13(24):6348. doi: 10.3390/cancers13246348 (PMC8699806; doi:10.3390/cancers13246348)
Supplement: Supplementary file 1 [file cancers-13-06348-s001.zip › cancers-1477818-supplementary.pdf]

Table S8: Differential methylation across promoter and body of TRGs in D2 subgroup

Gene	Chr	DMR Start	DMR End	Length	CpGs	Pr	Gene start
DDX1	2	15586861	15587037	177	7	0.001356	15586178
MTR	1	236792904	236792971	68	3	0.025082	236790281
RAD54L	1	46243621	46243753	133	5	0.009496	46242695
TERF1	8	73007342	73007540	199	8	0.026215	73003864
TP53BP1	15	43513051	43513250	200	8	0.00022	43510414
ZNF208	19	22012974	22013176	203	8	0.020887	22010749
ACYP2	2	54106744	54106878	135	6	0.000212	53971103
ACYP2	2	54150834	54150901	68	4	0	53971103
BICD1	12	32278625	32278711	87	4	0.009196	32107151
DDB1	11	61339448	61339661	214	4	0.001745	61300051
MAD1L1	7	2167473	2167653	181	7	4.30E-05	1815794
MAD1L1	7	2121852	2121927	76	3	0.049216	1815794
MAD1L1	7	2071592	2071734	143	4	0.008698	1815794
MAD1L1	7	1912925	1912988	64	5	0.00554	1815794
MAD1L1	7	1912819	1912924	106	7	0.031169	1815794
MAD1L1	7	1885830	1885906	77	1	0.006091	1815794
MEN1	11	64807039	64807137	99	1	0.009018	64803524
MSH2	2	47497533	47497630	98	3	0.003851	47403130
PARP1	1	226369232	226369389	158	4	0.003036	226360691
PIK3C3	18	42024964	42025106	143	6	0.00164	41955206
PML	15	74044424	74044501	78	2	0.021978	73994729
PRMT8	12	3414514	3414628	115	2	1.43E-07	3381349
PRMT8	12	3415880	3415981	102	2	3.32E-07	3381349
PRMT8	12	3421417	3421493	77	1	0.000735	3381349
PRMT8	12	3429748	3429815	68	2	0.002224	3381349
PRMT8	12	3435129	3435258	130	2	4.08E-05	3381349
PRMT8	12	3435614	3435764	151	2	0.011784	3381349
PRMT8	12	3435765	3435814	50	5	0.000161	3381349
PRMT8	12	3442751	3442951	201	6	2.92E-05	3381349
PRMT8	12	3456584	3456739	156	6	2.27E-05	3381349
PRMT8	12	3470681	3470732	52	2	2.29E-05	3381349
PRMT8	12	3472267	3472483	217	2	0.000719	3381349
PRMT8	12	3491088	3491127	40	6	1.05E-08	3381349
PRMT8	12	3491618	3491747	130	12	0.023697	3381349
PRMT8	12	3491748	3491821	74	11	0.000556	3381349
PRMT8	12	3507214	3507281	68	5	0.000297	3381349
PRMT8	12	3508414	3508500	87	3	0.047165	3381349
PRMT8	12	3508501	3508672	172	5	0.000905	3381349
PRMT8	12	3508673	3508829	157	6	0.00017	3381349
PRMT8	12	3531734	3531852	119	4	0.000581	3381349
PRMT8	12	3544694	3544907	214	6	0.000139	3381349
PRMT8	12	3551337	3551507	171	4	5.02E-10	3381349
PRMT8	12	3551508	3551560	53	2	4.70E-08	3381349
PRMT8	12	3551561	3551708	148	6	1.51E-07	3381349
PRMT8	12	3552084	3552150	67	3	1.18E-08	3381349
PRMT8	12	3569504	3569585	82	3	3.88E-05	3381349
PRMT8	12	3576909	3577001	93	3	0.00011	3381349
PRMT8	12	3577002	3577060	59	2	5.64E-08	3381349

PRMT8	12	3579345	3579497	153	4	4.81E-08	3381349
PRMT8	12	3592296	3592425	130	4	3.00E-05	3381349
RAD51	15	40727952	40728019	68	3	0.003545	40695160
RAP1A	1	111542789	111542995	207	3	0.000808	111542218
RAP1A	1	111582907	111583087	181	1	0.011713	111542218
RAP1A	1	111608060	111608115	56	5	0.001017	111542218
RAP1A	1	111689762	111689836	75	5	0.015864	111542218
RECQL4	8	144516607	144516760	154	3	0.02581	144511288
RECQL4	8	144514151	144514213	63	4	0.000214	144511288
RECQL5	17	75633383	75633484	102	3	0.000587	75626854
RTEL1	20	63680763	63680980	218	5	0.042192	63657810
RTEL1	20	63686672	63686770	99	6	0.032388	63657810
TERF2	16	69405138	69405341	204	5	0.000158	69372269
TERT	5	1294255	1294331	77	8	0.031573	1253167
TP53BP1	15	43444429	43444554	126	1	2.42E-06	43407209
ZNF208	19	22009907	22010108	202	15	0.02821	21932958
ZNF208	19	22008159	22008226	68	1	0.001737	21932958
ZNF208	19	22008026	22008158	133	5	7.92E-06	21932958
ZNF208	19	21998451	21998527	77	1	8.28E-05	21932958
ZNF208	19	21998315	21998450	136	6	2.06E-05	21932958
ZNF208	19	21940986	21941044	59	5	2.09E-08	21932958
ZNF208	19	21940729	21940933	205	11	0.000335	21932958

Abbreviations: Chr, chromosome; CpGs, cytosine and guanine occuring consecutively; Diff Meth, differential metl

Gene stop	gene_transcript_id	Position	gene_strand	gene_TSS	Control
15591378	ENST00000381341.6	2:15586178-15591378	+	15591178	0.9423
236795481	ENST00000535889.5	1:236790281-236795481	+	236795281	0.7863
46247895	ENST00000442598.5	1:46242695-46247895	+	46247695	0.7973
73009064	ENST00000276602.10	8:73003864-73009064	+	73008864	0.9268
43515614	ENST00000263801.7	15:43510414-43515614	-	43510614	0.7143
22015949	ENST00000599916.5	19:22010749-22015949	-	22010949	0.8182
54304859	ENST00000607452.5	2:53971103-54304859	+	53971103	0.5049
54304859	ENST00000607452.5	2:53971103-54304859	+	53971103	1
32383633	ENST00000548411.5	12:32107151-32383633	+	32107151	0.7955
61342596	ENST00000540166.5	11:61300051-61342596	-	61342596	0.8571
2233243	ENST00000406869.5	7:1815794-2233243	-	2233243	0.875
2233243	ENST00000406869.5	7:1815794-2233243	-	2233243	0.8399
2233243	ENST00000406869.5	7:1815794-2233243	-	2233243	0.3822
2233243	ENST00000406869.5	7:1815794-2233243	-	2233243	0.2804
2233243	ENST00000406869.5	7:1815794-2233243	-	2233243	0.3446
2233243	ENST00000406869.5	7:1815794-2233243	-	2233243	0.6591
64811294	ENST00000337652.5	11:64803524-64811294	-	64811294	0.6667
47513234	ENST00000406134.5	2:47403130-47513234	+	47403130	0.5294
226408073	ENST00000366794.9	1:226360691-226408073	-	226408073	0.0915
42087830	ENST00000262039.8	18:41955206-42087830	+	41955206	0.4205
74047812	ENST00000565898.5	15:73994729-74047812	+	73994729	0.674
3593182	ENST00000452611.6	12:3381349-3593182	+	3381349	0.7341
3593182	ENST00000452611.6	12:3381349-3593182	+	3381349	0.6402
3593182	ENST00000452611.6	12:3381349-3593182	+	3381349	0.5789
3593182	ENST00000452611.6	12:3381349-3593182	+	3381349	0.4713
3593182	ENST00000452611.6	12:3381349-3593182	+	3381349	0.6061
3593182	ENST00000452611.6	12:3381349-3593182	+	3381349	0.4
3593182	ENST00000452611.6	12:3381349-3593182	+	3381349	0.7917
3593182	ENST00000452611.6	12:3381349-3593182	+	3381349	0.4605
3593182	ENST00000452611.6	12:3381349-3593182	+	3381349	0.6766
3593182	ENST00000452611.6	12:3381349-3593182	+	3381349	0.5281
3593182	ENST00000452611.6	12:3381349-3593182	+	3381349	0.8
3593182	ENST00000452611.6	12:3381349-3593182	+	3381349	0.2944
3593182	ENST00000452611.6	12:3381349-3593182	+	3381349	0.2992
3593182	ENST00000452611.6	12:3381349-3593182	+	3381349	0.3447
3593182	ENST00000452611.6	12:3381349-3593182	+	3381349	0.728
3593182	ENST00000452611.6	12:3381349-3593182	+	3381349	0.8326
3593182	ENST00000452611.6	12:3381349-3593182	+	3381349	0.4828
3593182	ENST00000452611.6	12:3381349-3593182	+	3381349	0.7137
3593182	ENST00000452611.6	12:3381349-3593182	+	3381349	0.8879
3593182	ENST00000452611.6	12:3381349-3593182	+	3381349	0.8571
3593182	ENST00000452611.6	12:3381349-3593182	+	3381349	0.7771
3593182	ENST00000452611.6	12:3381349-3593182	+	3381349	0.5867
3593182	ENST00000452611.6	12:3381349-3593182	+	3381349	0.7416
3593182	ENST00000452611.6	12:3381349-3593182	+	3381349	0.7049
3593182	ENST00000452611.6	12:3381349-3593182	+	3381349	0.8344
3593182	ENST00000452611.6	12:3381349-3593182	+	3381349	0.4573
3593182	ENST00000452611.6	12:3381349-3593182	+	3381349	0.5361

3593182	ENST00000452611.6	12:3381349-3593182	+	3381349	0.6615
3593182	ENST00000452611.6	12:3381349-3593182	+	3381349	0.6907
40732339	ENST00000267868.7	15:40695160-40732339	+	40695160	1
111713113	ENST00000356415.5	1:111542218-111713113	+	111542218	0.8736
111713113	ENST00000356415.5	1:111542218-111713113	+	111542218	0.7556
111713113	ENST00000356415.5	1:111542218-111713113	+	111542218	0.6883
111713113	ENST00000356415.5	1:111542218-111713113	+	111542218	0.055
144517845	ENST00000621189.4	8:144511288-144517845	-	144517845	0.6939
144517845	ENST00000621189.4	8:144511288-144517845	-	144517845	0.0495
75667189	ENST00000317905.9	17:75626854-75667189	-	75667189	0.5951
63696253	ENST00000370018.7	20:63657810-63696253	+	63657810	0.7186
63696253	ENST00000370018.7	20:63657810-63696253	+	63657810	0.3282
69408571	ENST00000567841.1	16:69372269-69408571	-	69408571	0.7561
1295047	ENST00000310581.9	5:1253167-1295047	-	1295047	0.4422
43510614	ENST00000263801.7	15:43407209-43510614	-	43510614	0.8947
22010949	ENST00000599916.5	19:21932958-22010949	-	22010949	0.4939
22010949	ENST00000599916.5	19:21932958-22010949	-	22010949	0.8696
22010949	ENST00000599916.5	19:21932958-22010949	-	22010949	0.6442
22010949	ENST00000599916.5	19:21932958-22010949	-	22010949	0.7714
22010949	ENST00000599916.5	19:21932958-22010949	-	22010949	0.6977
22010949	ENST00000599916.5	19:21932958-22010949	-	22010949	0.6615
22010949	ENST00000599916.5	19:21932958-22010949	-	22010949	0.5753

hylation; DMR; differentially methylated regions; NDMM, newly diagnosed multiple myeloma; Pr, probability; TRG, telo

D2	Diff Meth	Position	TSS distance (kb)
0.4205	-0.5218	Promoter	-4.141
0.3011	-0.4852	Promoter	-2.31
0.3459	-0.4514	Promoter	-3.942
0.5786	-0.3482	Promoter	-1.324
0.2857	-0.4286	Promoter	-2.437
0.4213	-0.3969	Promoter	-2.025
0.2137	-0.2912	GeneBody	135.641
0.5	-0.5	GeneBody	179.731
0.5824	-0.2131	GeneBody	171.474
0.5188	-0.3383	GeneBody	2.935
0.6667	-0.2083	GeneBody	65.59
0.6399	-0.2	GeneBody	111.316
0.634	0.2518	GeneBody	161.509
0.6242	0.3438	GeneBody	320.255
0.6923	0.3477	GeneBody	320.319
0.455	-0.2041	GeneBody	347.337
0.2586	-0.4081	GeneBody	4.157
0.8125	0.2831	GeneBody	94.403
0.2989	0.2074	GeneBody	38.684
0.74	0.3195	GeneBody	69.758
0.4651	-0.2089	GeneBody	49.695
0.2101	-0.524	GeneBody	33.165
0.2059	-0.4343	GeneBody	34.531
0.2449	-0.334	GeneBody	40.068
0.1846	-0.2867	GeneBody	48.399
0.2326	-0.3735	GeneBody	53.78
0.1481	-0.2519	GeneBody	54.265
0.2466	-0.5451	GeneBody	54.416
0.0657	-0.3948	GeneBody	61.402
0.3636	-0.313	GeneBody	75.235
0.1397	-0.3884	GeneBody	89.332
0.0769	-0.7231	GeneBody	90.918
0.0387	-0.2557	GeneBody	109.739
0.0918	-0.2074	GeneBody	110.269
0.1046	-0.2401	GeneBody	110.399
0.3665	-0.3615	GeneBody	125.865
0.5667	-0.2659	GeneBody	127.065
0.1667	-0.3161	GeneBody	127.152
0.2616	-0.4521	GeneBody	127.324
0.6343	-0.2536	GeneBody	150.385
0.3679	-0.4892	GeneBody	163.345
0.1274	-0.6497	GeneBody	169.988
0.1269	-0.4598	GeneBody	170.159
0.2447	-0.4969	GeneBody	170.212
0.2019	-0.503	GeneBody	170.735
0.3509	-0.4835	GeneBody	188.155
0.2186	-0.2387	GeneBody	195.56
0.1258	-0.4103	GeneBody	195.653

0.1119	-0.5496	GeneBody	197.996
0.2012	-0.4895	GeneBody	210.947
0.7143	-0.2857	GeneBody	32.792
0.4082	-0.4654	GeneBody	0.571
0.5	-0.2556	GeneBody	40.689
0.4581	-0.2302	GeneBody	65.842
0.2666	0.2116	GeneBody	147.544
0.901	0.2071	GeneBody	1.085
0.3594	0.3099	GeneBody	3.632
0.315	-0.2801	GeneBody	33.705
0.5184	-0.2002	GeneBody	22.953
0.5492	0.221	GeneBody	28.862
0.4198	-0.3363	GeneBody	3.23
0.7105	0.2683	GeneBody	0.716
0.6102	-0.2845	GeneBody	66.06
0.2836	-0.2103	GeneBody	0.841
0.5042	-0.3654	GeneBody	2.723
0.2052	-0.439	GeneBody	2.791
0.15	-0.6214	GeneBody	12.422
0.1343	-0.5634	GeneBody	12.499
0.1596	-0.5019	GeneBody	69.905
0.2225	-0.3528	GeneBody	70.016

mere-related gene; TSS, transcription start site
